# Rib fixation versus non-operative treatment for flail chest and multiple rib fractures after blunt thoracic trauma: a multicenter cohort study

**DOI:** 10.1007/s00068-018-1037-1

**Published:** 2018-10-19

**Authors:** Reinier B. Beks, David Reetz, Mirjam B. de Jong, Rolf H. H. Groenwold, Falco Hietbrink, Michael J. R. Edwards, Luke P. H. Leenen, Roderick Marijn Houwert, Jan Paul M. Frölke

**Affiliations:** 1 Department of Surgery, University Medical Center Utrecht, PO Box 85500, 3508 GA Utrecht, The Netherlands; 2 Utrecht Traumacenter, Utrecht, The Netherlands; 3 Department of Surgery, Radboud University Medical Center, Nijmegen, The Netherlands; 4 Julius Center for Health Sciences and Primary Care, UMC Utrecht, Utrecht, The Netherlands; 5 Department of Clinical Epidemiology, Leiden University Medical Center, Leiden, The Netherlands

**Keywords:** Rib fixation, Non-operative treatment, Flail chest, Multiple rib fractures, Rib fracture

## Abstract

**Background:**

Over the years, a trend has evolved towards operative treatment of flail chest although evidence is limited. Furthermore, little is known about operative treatment for patients with multiple rib fractures without a flail chest. The aim of this study was to compare rib fixation based on a clinical treatment algorithm with nonoperative treatment for both patients with a flail chest or multiple rib fractures.

**Methods:**

All patients with ≥ 3 rib fractures admitted to one of the two contributing hospitals between January 2014 and January 2017 were retrospectively included in this multicenter cohort study. One hospital treated all patients nonoperatively and the other hospital treated patients with rib fixation according to a clinical treatment algorithm. Primary outcome measures were intensive care length of stay and hospital length of stay for patients with a flail chest and patients with multiple rib fractures, respectively. To control for potential confounding, propensity score matching was applied.

**Results:**

A total of 332 patients were treated according to protocol and available for analysis. The mean age was 56 (SD 17) years old and 257 (77%) patients were male. The overall mean Injury Severity Score was 23 (SD 11) and the average number of rib fractures was 8 (SD 4). There were 92 patients with a flail chest, 37 (40%) had rib fixation and 55 (60%) had non-operative treatment. There were 240 patients with multiple rib fractures, 28 (12%) had rib fixation and 212 (88%) had non-operative treatment. For both patient groups, after propensity score matching, rib fixation was not associated with intensive care unit length of stay (for flail chest patients) nor with hospital length of stay (for multiple rib fracture patients), nor with the secondary outcome measures.

**Conclusion:**

No advantage could be demonstrated for operative fixation of rib fractures. Future studies are needed before rib fixation is embedded or abandoned in clinical practice.

## Background

Multiple rib fractures are the most common type of thoracic injury and are associated with a high morbidity and mortality, which is to a certain extent due to associated injuries [1–4]. Still, an increased number of rib fractures corresponds to a worse outcome, in part due to respiratory complications resulting from pain and an impaired ventilation capacity [5–7]. Consequently, superinfection leading to pneumonia and prolonged mechanical ventilation are common in patients with chest wall injuries [2]. It is important to distinguish between multiple rib fractures with and without a flail chest, as the latter is associated with an increased mortality rate and significant morbidity [8–11].

Nonoperative treatment has been the gold standard for the past few decades and is focused on the underlying pulmonary contusion- and rib fracture-associated complications, including pain, atelectasis, and compromised pulmonary hygiene [4]. Over the years, a trend has evolved towards operative treatment of flail chest as physicians aim to improve mortality rates and reduce the prolonged length of stay for these patients. In a recent systematic review, rib fixation in patients with a flail chest was associated with a reduced: intensive care unit length of stay, days on mechanical ventilation, mortality rate, pneumonia rate, and treatment costs, although evidence remains limited [12]. Studies investigating the effect of rib fixation in patients with multiple rib fractures are even more scarce, although two retrospective cohort studies showed promising results [13, 14].

For both flail chest and multiple rib fractures, the indication for surgery is heterogeneously described in the aforementioned studies [12–14]. Therefore, no clear consensus on indication is available based on the current literature. It can be hypothesized that for patients with multiple rib fractures, early fixation might be beneficial. Therefore, the aim of this study was to compare rib fixation based on a clinical treatment algorithm with nonoperative treatment for both patients with a flail chest and patients with multiple rib fractures.

## Methods

### Study design and participants

All patients with three or more rib fractures admitted to one of the two contributing hospitals between January 2014 and January 2017 were retrospectively included in this multicenter cohort study. Both hospitals are academic tertiary referral centers with a level one trauma facility of similar size. Patients were included if they fulfilled the following criteria: age 18 years and older, blunt thoracic trauma resulting in multiple rib fractures (defined as three or more rib fractures) or a flail chest (defined as three or more consecutive ribs fractured in at least two places and clinical signs of paradoxical chest wall movement), and being alive 2 days after hospital admission (mean time till surgery). Exclusion criteria were: transfer to another hospital, initial admission in another hospital, no availability of a computed tomography (CT) scan, and rib fixation more than 4 days after trauma. Patients were followed from admission until discharge or death.

**Fig. 1:**
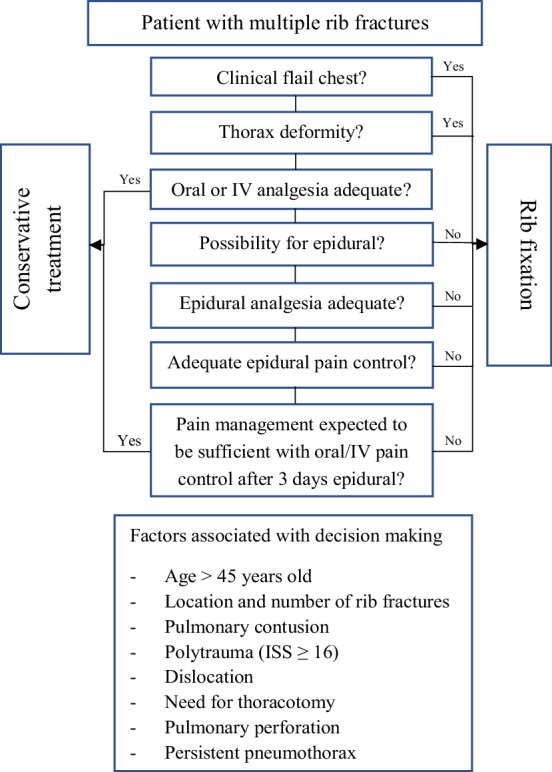
Clinical treatment algorithm for patients with rib fractures

Eligible patients were identified using procedural codes and the Dutch National Trauma Registry. The non-operative group was formed by all patients with rib fractures admitted to the Radboud University Medical Center where treatment consisted of adequate pain management, supportive mechanical ventilation when indicated, and physiotherapy for breathing exercises according to standard national guidelines. Per protocol every patient with three or more rib fractures was considered for epidural analgesia, if needed this was supported by patient-controlled anesthesia (intravenous opioids). Epidural therapy was provided between days 1–5 after trauma. The epidural was removed after 5 days in situ due to the considered risk for infection. The surgical group consisted of all patients who had rib fixation performed in the University Medical Center Utrecht where the same non-operative treatment guidelines were followed, but in addition, rib fixation was considered according to a clinical-based algorithm (Fig. 1). Pain was arbitrarily defined as a numerical rating scale of 5 or higher during coughing or deep inspiration and if pain was suspected not to decrease over the subsequent days with adequate pain management. It was the subjective decision of the surgeon-on-call to perform rib fixation.

This study was approved by the institutional review board of the participating centers (METC 17–544/C and 2016–2861).

### Surgical procedure rib fixation

All procedures were performed by one of the even senior trauma surgeons experienced in surgical treatment of rib fractures. Rib fixation is performed in this center since 2006. Preoperative planning of the procedure was done using chest computed tomography (CT) with 3D reconstructions. Preoperative antibiotic prophylaxis (2 g of Cefazolin) was administered intravenously in all patients. Depending on the site of the fractures, patients were positioned in the supine, lateral or prone position and the surgical approach was performed as described by Taylor [15]. In the case of intercostal muscle interposition, debridement was performed. After reduction, internal fixation using the MatrixRIB™ system (Depuy Synthes®, Amersfoort, The Netherlands) was performed. Fixation was preferably done with three bicortical screws on each side of the fracture. The number of fixed ribs was at the discretion of the surgeon, and depended upon the possibility to regain stability of the chest wall during respirations. Tube thoracostomy was only performed in the case of clinical suspicion of pneumothorax during surgery. Postoperative chest radiography was performed in all patients to document surgical result and to rule out complications. Patients were allowed to perform their daily activities as soon as possible.

### Baseline characteristics

Data for the following baseline characteristics were extracted from medical records: age, sex, American Society of Anesthesiologists (ASA) score, trauma mechanism, Injury Severity Score (ISS), thoracic trauma severity score (TTSS), abbreviated injury scale (AIS) head, AIS face, AIS thorax, AIS abdomen, AIS extremities, number of rib fractures, bilateral rib fractures, concomitant injuries (pulmonary contusion, pneumothorax, hemothorax, sternum fracture) as recorded on the admission CT scan, and first available blood pH and base excess; additionally, for the surgical group: duration until surgery in days, duration of surgery in minutes, and number of surgically-fixated rib fractures. The ISS is a measure (range 0–75) of the severity of traumatic injury and is calculated by adding the square of the three highest AIS scores. The AIS is a standardized anatomical-based coding system ranging from 0 to 5 to classify the severity of traumatic injury per body region. The AIS is registered in the Dutch National Trauma Registry by trained data managers based on radiology reports from admission CT scans and medical records. The TTSS is a score (range 0–25) based on number of rib fractures, pulmonary contusion, PaO_2_/FiO_2_ ratio, age, and pleural involvement, and helps to predict outcome after thoracic trauma [16–18]. Rib fractures, pulmonary contusion, pneumothorax, and hemothorax were assessed on the admission CT scan.

### Outcome measures

In line with previous trial reports, the primary outcome measure for patients with a flail chest was intensive care unit length of stay (ILOS) and for patients with multiple rib fractures, and hospital length of stay (HLOS). For both patient groups, secondary outcome measures were duration of invasive mechanical ventilation (IMV), duration of epidural analgesia, pneumonia, need for tracheostomy and in hospital mortality. Pneumonia was defined as having clinical signs (fever, coughing, desaturation) requiring antibiotic treatment, with or without positive cultures. Additionally, we assessed in hospital complications after rib fixation.

### Statistical analysis

All analyses were stratified by patient group, i.e., performed separately for patients with a flail chest and patients with multiple rib fractures. Baseline characteristics were presented as proportions for categorical variables, mean and standard deviation (SD) for normally distributed continuous variables, and median and interquartile range (IQR) for non-normally distributed continuous variables. Differences in distributions of baseline characteristics between the study groups were quantified by means of standardized differences and statistical tests (t-test for normally distributed continuous data, Mann–Whitney test for non-normally distributed data, and chi-square test for categorical data) [19].

We applied multiple imputation (25 times) to impute missing values for ASA [2.1% (7/332)], TTSS [20% (67/332)], AIS head [0.6% (2/332)], pulmonary contusion [0.6% (2/332)], pH [9.0% (30/332)], and base excess [9.0% (30/332)]. Multiple imputation was performed using the mice() algorithm in R [20].

To control for potential confounding, propensity score (PS) matching was applied. To minimize the effects of selection bias, we matched patients from the ‘operating’ center with patients from the ‘nonoperative’ center, based on all baseline characteristics. First, a PS model was fitted using logistic regression analysis, with rib fracture fixation as the dependent variable, and age, sex, ASA-score, trauma mechanism, ISS, TTSS, AIS head, AIS face, AIS thorax, AIS abdomen, AIS extremities, number of rib fractures, bilateral rib fractures, concomitant injuries, blood pH, and base excess were included as covariates in the model. We performed 2:1 nearest neighbor matching, with a maximum caliper of 0.2 of the standard deviation of the logit of the PS using the Matchit() algorithm in R [21]. After matching, the balance in the distributions of baseline characteristics between the study groups was quantified using standardized differences, where a standardized difference < 0.1 is generally accepted as indicating fair balance of confounders between the matched treatment groups (i.e., successful matching) [19].

In the primary analysis, for patients with a flail chest, we estimated the relation between rib fracture fixation and ILOS by means of linear regression analysis. For patients with multiple rib fractures, we estimated the relation between rib fracture fixation and HLOS by means of linear regression analysis. Secondary analyses focused on the relation of rib fracture fixation with duration of IMV and duration of epidural analgesia using linear regression analysis. The relation between rib fixation, pneumonia, tracheostomy, and in hospital mortality was assessed by means of a logistic regression analysis.

A two-tailed *p* value less than 0.05 was considered significant. All analyses were performed using R v3.4.1 [22].

## Results

**Fig. 2:**
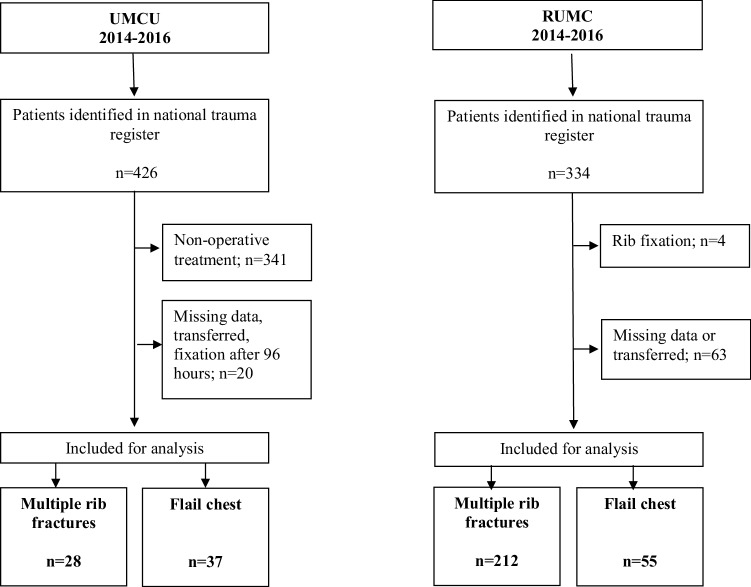
Flowchart showing inclusion of patients for analysis. “UMCU” University Medical Center Utrecht; “RUMC” Radboud University Medical Center

A total of 332 patients were available for analysis (Fig. 2). The overall mean age was 56 (SD 17) years old and 257 (77%) patients were male (Table 1). Most patients were injured in a motor vehicle accident or after a fall from height resulting on average in 8 (SD 4) rib fractures and an overall mean ISS of 23 (SD 11).

**Table 1:** Baseline characteristics of patients with flail chest or multiple rib fractures before propensity score matching

Variable	Multiple rib fractures					Flail chest				
	Surgery (*n* = 28)	Non-operative (*n* = 212)	*p* value	SMD unmatched cohort	SMD matched cohort	Surgery (*n* = 37)	Non-operative (*n* = 55)	*p* value	SMD unmatched cohort	SMD matched cohort
Age (mean ± SD)										
Male (n, %)										
ASA-score (n, %)			0.363	0.270	0.040			0.195	0.452	0.029
1–2	26 (93)	179 (84)				37 (100)	50 (91)			
> 2	2 (7)	33 (16)				0 (0)	5 (9)			
Trauma mechanism (*n*, %)			0.661	0.181	0.081			0.439	0.273	0.175
Motor vehicle accident	8 (29)	74 (35)				14 (38)	14 (26)			
Fall from height/stairs	11 (39)	66 (31)				10 (27)	19 (35)			
Other	9 (32)	72 (34)				13 (35)	22 (40)			
ISS (mean ± SD)	21 (9.4)	21 (9.6)	0.933	0.017	0.077	25 (12)	30 (13)	0.054	0.418	0.050
TTSS (mean ± SD)	10 (3.6)	9.3 (2.9)	0.237	0.242	0.101	13.1 (3.1)	13 (2.9)	0.951	0.014	0.087
AIS (median, IQR)										
Head	0 (0–1)	1 (0–3)	**0.008**	0.576	0.088	0 (0–3)	3 (0.3–4)	**0.001**	0.722	0.050
Face	0 (0–0)	0 (0–0)	0.138	0.269	0.053	0 (0–1)	0 (0–0.5)	0.796	0.077	0.069
Thorax	3 (3–4)	3 (3–3)	**0.010**	0.567	0.017	3 (3–4)	4 (3–4)	0.149	0.301	0.102
Abdomen	2 (0–2)	0 (0–0)	< **0.001**	0.585	0.016	0 (0–2)	0 (0–0.5)	0.238	0.244	0.118
Extremities	1 (0–2.3)	2 (0–2)	0.770	0.079	0.040	2 (0–3)	2 (0–3)	0.442	0.164	0.097
No. of rib fractures (median, IQR)	7 (6–10.3)	6 (4–8)	**0.001**	0.643	0.096	10 (8–12)	9 (7–13)	0.405	0.049	0.059
Bilateral rib fractures (n, %)	10 (36)	69 (33)	0.904	0.067	0.041	14 (38)	25 (46)	0.610	0.155	0.037
Concomitant injuries (n, %)										
Pulmonary contusion	11 (39)	99 (47)	0.561	0.159	0.017	22 (60)	38 (69)	0.467	0.202	0.094
Pneumothorax	19 (68)	117 (55)	0.285	0.263	0.058	27 (73)	47 (86)	0.226	0.311	0.071
Hemothorax	3 (11)	37 (18)	0.529	0.195	0.059	9 (24)	22 (40)	0.182	0.340	0.041
Sternum fracture	5 (18)	24 (11)	0.491	0.186	0.071	5 (14)	9 (16)	0.938	0.080	0.053
Blood pH (mean ± SD)	7.34 (0.12)	7.32 (0.08)	0.308	0.173	0.046	7.33 (0.1)	7.27 (0.13)	0.034	0.475	0.036
Base excess (mean ± SD)	− 2.4 (5.6)	− 3.1 (3.5)	0.367	0.154	0.052	− 2.6 (3.5)	− 4.7 (6.2)	0.063	0.426	0.068

Bold values indicate significant *p* values (< 0.05)

*SMD* standardized mean difference; *ASA* American Society of Anesthesiologists; *ISS* injury severity score; *TTSS* thoracic trauma severity score; *AIS* abbreviated injury score; *SD* standard deviation; *IQR* interquartile range

Of the 92 patients with a flail chest, 37 (40%) had rib fixation and 55 (60%) had non-operative treatment (Fig. 2). For the flail chest population, surgically treated patients had a lower AIS head and a higher blood pH. Among the 240 patients with multiple rib fractures, 28 (12%) had rib fixation and 212 (88%) had non-operative treatment. In this group, surgical patients had a significantly lower AIS head, higher AIS thorax, higher AIS abdomen, and higher number of rib fractures (Table 1).

The median time until surgery was 1 day (IQR 1–2) (Table 2). The median number of surgically-fixated rib fractures for patients with a flail chest was 5 (4–6) and for patients with multiple rib fractures was 4 (IQR 3–5). Four (6%) patients were treated with both plate osteosynthesis and intramedullary splints; two patients with flail chest and two with multiple rib fractures. Nine (14%) patients had a postoperative complication. Two patients had a persistent postoperative pneumothorax and were treated with a chest tube. Two patients developed pleural empyema requiring video-assisted thoracoscopic surgery to evacuate the empyema. One patient had a postoperative tension pneumothorax and was treated with a chest tube. One patient had a hemothorax and required a thoracotomy to evacuate the hematoma. One patient had excess pleural fluid and was treated with a chest tube. One patient had a hematoma near the surgical incision and needed surgical debridement of the old hematoma. And one patient had a deep infection near the osteosynthesis material and was successfully treated with antibiotics.

**Table 2:** Surgery-related characteristics and in-hospital complications

Variables	Multiple rib fracture	Flail chest
	*n* = 28	*n* = 37
Duration until rib fixation in days (median, IQR)	1 (1–2)	1 (1–2)
Duration of surgery in minutes (mean ± SD)	130 (83)	148 (64)
Number of surgically-fixated rib fractures (median, IQR)	4 (3–5)	5 (4–6)
Ratio surgically-fixated ribs and total number of rib fractures	0.54	0.50
In-hospital complications after surgical rib fixation (*n*, %)		
Pneumothorax	0 (0)	2 (5.4)
Tension pneumothorax	1 (3.6)	0 (0)
Pleural empyema	0 (0)	2 (5.4)
Excess pleural fluid	1 (3.6)	0 (0)
Infection of osteosynthesis material	1 (3.6)	0 (0)
Hematoma	0 (0)	1 (2.7)
Hemothorax	0 (0)	1 (2.7)

After propensity score matching, for patients with a flail chest there was no association of rib fixation and ILOS [confidence interval (CI) − 13.9 to 8.5, *p* = 0.638] and the secondary outcome measures (Table 3). For patients with multiple rib fractures, there was no association between rib fixation and HLOS (CI − 0.6 to 13.6, *p* = 0.074) and the secondary outcome measures (Table 4).

**Table 3:** Regression analysis assessing the influence of rib fixation for a flail chest after propensity score matching

Continuous variables	Rib fixation for flail chest					
	Median (IQR)		*δ*	95% CI	SE	*p* value
	Surgery	Non-operative				
Duration of ICU stay in days	6 (0–13)	2 (0–8)	− 2.7	− 13.9 to 8.5	5.721	0.638
Duration of IMV in days	3 (0–9)	0 (0–7)	− 2.3	− 11.6 to 7.0	4.750	0.624
Duration of epidural analgesia in days	0 (0–3)	2 (0–7)	− 1.2	− 3.4 to 1.0	1.116	0.290
Duration of hospital stay in days	21 (11–31)	11 (8–18)	1.9	− 14.3 to 18.0	8.240	0.820

	*n* (%)		OR	95% CI	SE	*p* value
Pneumonia	4.8 (23)	5.6 (20)	1.1	0.2 to 5.8	0.826	0.871
Tracheostomy	2.6 (12)	3.5 (13)	NA	NA to NA	NA	NA
In hospital mortality	2.2 (10)	3.3 (12)	NA	NA to NA	NA	NA

*SE* standard error; *OR* odds ratio; *ICU* intensive care unit; *IMV* invasive mechanical ventilation; *CI* confidence interval; *IQR* interquartile range; *NA* no answer

*δ* indicates the difference in mean outcome value between rib fixation and non-operative treatment

**Table 4:** Regression analysis assessing the influence of rib fixation for multiple rib fractures after propensity score matching

Outcome variable	Rib fixation for multiple rib fractures					
	Median (IQR)		*δ*	95% CI	SE	p value
	Surgery	Non-operative				
Duration of ICU stay in days	0 (0–11)	1 (0–2)	1.6	− 3.5 to 6.7	2.600	0.530
Duration of IMV in days	0 (0–9)	0 (0–1)	2.4	− 2.8 to 7.6	2.637	0.365
Duration of epidural analgesia in days	0 (0–4)	0 (0–3)	− 0.1	− 1.9 to 1.7	0.917	0.939
Duration of hospital stay in days	12 (9–23)	10 (6–16)	6.5	− 0.6 to 13.6	3.636	0.074

	*n* (%)		OR	95% CI	SE	*p* value
Pneumonia	7.4 (34)	5 (14)	3.2	0.8 to 13.9	0.743	0.114
Tracheostomy	1.7 (7.8)	0.7 (2)	NA	NA to NA	NA	NA
In-hospital mortality	0 (0)	3.3 (9.1)	NA	NA to NA	NA	NA

*SE* standard error; *OR* odds ratio; *ICU* intensive care unit; *IMV* invasive mechanical ventilation; *CI* confidence interval; *IQR* interquartile range; *NA* no answer

*δ* indicates the difference in mean outcome value between rib fixation and nonoperative treatment

## Discussion

We compared rib fixation with nonoperative treatment for both flail chest and multiple rib fractures. After propensity score matching, adjusting for all anticipated confounding variables, rib fixation for a flail chest was not associated with differences in ILOS or the other outcome measures. Neither did we find a difference in HLOS for rib fixation in patients with multiple rib fractures, nor for the other outcome measures.

In our study, there was no association between rib fixation and the primary and secondary outcome measures compared to nonoperative treatment for patients with a flail chest. Three RCTs have been published on this subject. The first was from Tanaka et al. who studied 37 patients (18 surgical, 19 non-operative) with a flail chest unable to wean from mechanical ventilation and performed surgery on average 7 days after admission; they excluded patients with severe head trauma, spinal injury, and no development of respiratory failure [23]. Granetzny et al. compared 40 patients (20 surgical, 20 non-operative) with a flail chest and performed surgery 24–36 h after intensive care admission; they excluded patients with disturbed consciousness after head trauma, fractures of the upper three ribs, and severe associated trauma to other systems [24]. Marasco et al. studied 46 patients (23 surgical, 23 non-operative) with a flail chest who were ventilator dependent without prospect of successful weaning within 48 h and performed surgery on average 4.6 days after admission; they excluded patients of 80 years old and older, spinal injury, open fractures, and a Glasgow Coma Scale of < 10 at the scene or on admission [25]. All three studies reported a significant decrease in DMV and ILOS. One possible explanation for these contrasting results compared to our study might be the more restrictive inclusion criteria used in the aforementioned studies. In our study, all patients with multiple rib fractures or a flail chest were studied, including patients with head trauma or other severe injuries. Less strict inclusion criteria will result in a more diverse patient selection and will increase the generalizability of the results; however, it could also have diminished the effect of rib fixation in an already heterogeneous patient group.

Interestingly, the ILOS of both the surgical (median 6 days; mean 8.9 days) and non-operative groups (median 3 days; mean 10.5 days) in our cohort were lower compared to Tanaka et al. (surgical: 16.5; non-operative: 26.8 days), Granetzny et al. (surgical: 9.6 and non-operative: 14.6 days), and Marasco et al. (surgical: 13.5 and non-operative: 18.7 days) [23–25]. Also the DMV in our entire cohort was lower compared to the published RCTs.

In the current literature, only one study compared rib fixation with non-operative treatment for patients with multiple rib fractures without a flail chest. In a retrospective study with 124 patients, Qiu et al. reported a significantly shorter HLOS after rib fixation for multiple rib fractures compared to non-operative treatment (11.1 days vs 15.9 days; *p* = 0.013) and also found lower pneumonia rates (4.6% vs 17%; *p* = 0.025) [13]. Fitzgerald et al. performed a cohort study of patients 65 years old and older with more than one rib fracture, but did not report the number of patients with a flail chest [14]. In that study, rib fixation resulted in a decrease in mortality and respiratory complications compared to non-operative treatment. Khandelwal et al. presented a study with 67 patients (38 surgical, 29 non-operative) with only two patients with a flail chest in the surgical group [26]. They found a significant reduction in pain intensity and early return to work after rib fixation.

Few studies have reported on complication rates after rib fixation. Of the published trials, only Granetzny et al. reported a complication rate of 35% including pneumonia and mortality [24]. Other complications were empyema (5%), mediastinitis (10%), wound infection (10%), and chest wall deformity (5%). In another prospective study, Pieracci et al. reported an infection rate of 3% after rib fixation but did not report on other complications [27]. In our study, nine (14%) of the surgically treated patients had a postoperative complication.

The results of this study should be interpreted considering several limitations. The retrospective design of the study might have affected the outcome measures due to the effects of data loss and under reporting. Although a clinical algorithm was used to select suitable patients for surgical treatment in UMCU, the final decision was made by the attending surgeon-on-call which is a potential selection bias. Pain is the most important indication for rib fixation in our clinical-based treatment algorithm. However, due to the retrospective design of this study, we were unable to compare pain scores and interventions for pain treatment. Therefore, we might have missed this potential beneficial effect of rib fixation. Instead, we used HLOS as a surrogate marker for treatment success, but this outcome measure might have been influenced by other factors such as intensive care treatment, ventilation modalities and logistic issues with patient transfer and could, therefore, have diminished differences in treatment effect. Additionally, we did not use a scoring system or other determinant for rib fixation other than the clinical algorithm (Fig. 1). However, we are confident to have included all the potential factors associated with decision-making in the operating center in our propensity score matched model. Finally, there is still no good fracture classification to distinguish between fracture type and location. It is speculated that lateral and lower rib fractures are more painful due to increased mobility of the fracture parts. Fracture classification could influence success of rib fixation and this should be investigated in future studies.

Even though this study is one of the largest studies reporting on this subject, the number of included patients is still relatively small and was possibly too small to detect relatively small yet clinically meaningful differences. Furthermore, as part of the between-hospital comparison and due to clinical practice, there were differences in the baseline criteria between the surgical group and the non-operatively treated group. However, using a propensity score model, we were able to successfully match on all measured baseline characteristics eliminating possible confounding due to measured patient characteristics. As with any observational study, our results are potentially biased by unmeasured confounding (e.g., subjective indications for surgery, pain scores and fracture classification), be it that we believe we have included most confounders in our analysis and the potential impact of unmeasured confounding therefore seems limited.

The University Medical Hospital Utrecht was the first hospital in the Netherlands to perform rib fixation for patients with flail chest and multiple rib fractures. With more than 7 years of experience, rib fixation has become an established procedure with a univocal clinical-based treatment algorithm, with its main focus on clinical signs of flail chest and pain. Nevertheless, no benefit could be demonstrated in this population with rib fractures who received early operative fixation in their clinical course. Therefore, results of this study, combined with the limited existing evidence and the substantial costs of surgical treatment, emphasize the need for future studies before rib fixation is embedded or abandoned in clinical practice, but also to identify specific patient groups who would benefit from rib fixation. These studies should focus on optimization of the indication and describe long-term outcome after rib fixation.

## References

[1] Bulger EM, Arneson MA, Mock CN, Jurkovich GJ (2000). Rib fractures in the elderly. J Trauma.

[2] Ziegler DW, Agarwal NN (1994). The morbidity and mortality of rib fractures. J Trauma.

[3] Lin FC-F, Li R-Y, Tung Y-W, Jeng K-C, Tsai SC-S (2016). Morbidity, mortality, associated injuries, and management of traumatic rib fractures. J Chin Med Assoc.

[4] Vana PG, Neubauer DC, Luchette FA (2014). Contemporary management of flail chest. Am Surg.

[5] Testerman GM (2006). Adverse outcomes in younger rib fracture patients. South Med J.

[6] Brasel K, Guse C, Layde P, Weigelt J (2006). Rib fractures: relationship with pneumonia and mortality. Crit Care Med.

[7] Flagel BT, Luchette FA, Reed RL, Esposito TJ, Davis KA, Santaniello JM (2005). Half-a-dozen ribs: the breakpoint for mortality. Surgery.

[8] Cannon RM, Smith JW, Franklin GA, Harbrecht BG, Miller FB, Richardson JD (2012). Flail chest injury: are we making any progress?. Am Surg.

[9] Dehghan N, De Mestral C, McKee MD, Schemitsch EH, Nathens A (2014). Flail chest injuries: a review of outcomes and treatment practices from the national trauma data bank. J Trauma Acute Care Surg.

[10] Liman ST, Kuzucu A, Tastepe AI, Ulasan GN, Topcu S (2003). Chest injury due to blunt trauma. Eur J Cardiothorac Surg.

[11] Clark GC, Schecter WP, Trunkey DD (1988). Variables affecting outcome in blunt chest trauma: flail chest vs. pulmonary contusion. J Trauma.

[12] Kasotakis G, Hasenboehler EA, Streib EW, Patel N, Patel MB, Alarcon L (2017). Operative fixation of rib fractures after blunt trauma: a practice management guideline from the Eastern Assocaiation for the Surgery of Trauma. J Trauma Acute Care Surg.

[13] Qiu M, Shi Z, Xiao J, Zhang X, Ling S, Ling H (2016). Potential benefits of rib fracture fixation in patients with flail chest and multiple non-flail rib fractures. Indian J Surg.

[14] Fitzgerald MT, Ashley DW, Abukhdeir H, Christie DB (2017). Rib fracture fixation in the 65 years and older population: a paradigm shift in management strategy at a Level I trauma center. J Trauma Acute Care Surg.

[15] Taylor BC, French BG, Fowler TT (2013). Surgical approaches for rib fracture fixation. J Orthop Trauma.

[16] Pape HC, Remmers D, Rice J, Evisch M, Krettek C, Tscherne H (2000). Appraisal of early evaluation of blunt chest trauma: development of a standardized scoring system for initial clinical decision making. J Trauma.

[17] Aukema TS, Beenen LFM, Hietbrink F, Leenen LPH (2011). Validation of the thorax trauma severity score for mortality and its value for the development of acute respiratory distress syndrome. Open Access Emerg Med.

[18] Martinez Casas I, Amador Marchante MA, Paduraru M, Fabregues Olea AI, Nolasco A, Medina JC (2016). Thorax trauma severity score: Is it reliable for patient’s evaluation in a secondary level hospital?. Bull Emerg Trauma.

[19] Austin PC (2011). An Introduction to propensity score methods for reducing the effects of confounding in observational studies. Multivariate Behav Res.

[20] van Buuren S, Groothuis-Oudshoorn K (2011). Mice: multivariate imputation by chained equations in *R*. J Stat Softw.

[21] Ho DE, Imai K, King G, Stuart EA, MatchIt (2011). Nonparametric preprocessing for parametric causal inference. J Stat Softw.

[22] R Core Team (2017). R: A Language and Environment for Statistical Computing.

[23] Tanaka H, Yukioka T, Yamaguti Y, Shimizu S, Goto H, Matsuda H (2002). Surgical stabilization of internal pneumatic stabilization? A prospective randomized study of management of severe flail chest patients. J Trauma.

[24] Granetzny A, Abd El-Aal M, Emam E, Shalaby A, Boseila A (2005). Surgical versus conservative treatment of flail chest. Evaluation of the pulmonary status. Interact Cardiovasc Thorac Surg.

[25] Marasco SF, Davies AR, Cooper J, Varma D, Bennett V, Nevill R (2013). Prospective randomized controlled trial of operative rib fixation in traumatic flail chest. J Am Coll Surg.

[26] Khandelwal G, Mathur RK, Shukla S, Maheshwari A (2011). A prospective single center study to assess the impact of surgical stabilization in patients with rib fracture. Int J Surg.

[27] Pieracci FM, Lin Y, Rodil M, Synder M, Herbert B, Tran DK (2016). A prospective, controlled clinical evaluation of surgical stabilization of severe rib fractures. J Trauma Acute Care Surg.

